# Knee kinematics and kinetics in former soccer players with a 16-year-old ACL injury – the effects of twelve weeks of knee-specific training

**DOI:** 10.1186/1471-2474-8-35

**Published:** 2007-04-17

**Authors:** Anette von Porat, Marketta Henriksson, Eva Holmström, Ewa M Roos

**Affiliations:** 1 Department of Health Sciences, Division of Physiotherapy, Lund University, Lund, Sweden; 2 Department of Neurobiology, Care Sciences and Society, Karolinska Institutet, Stockholm, Sweden; 3 Department of Orthopaedics, Clinical Sciences Lund, Lund University, Sweden

## Abstract

**Background:**

Training of neuromuscular control has become increasingly important and plays a major role in rehabilitation of subjects with an injury to the anterior cruciate ligament (ACL). Little is known, however, of the influence of this training on knee stiffness during loading. Increased knee stiffness occurs as a loading strategy of ACL-injured subjects and is associated with increased joint contact forces. Increased or altered joint loads contribute to the development of osteoarthritis.

The aim of the study was to determine if knee stiffness, defined by changes in knee kinetics and kinematics of gait, step activity and cross-over hop could be reduced through a knee-specific 12-week training programme.

**Methods:**

A 3-dimensional motion analysis system (VICON) and a force plate (AMTI) were used to calculate knee kinetics and kinematics before and after 12 weeks of knee-specific training in 12 males recruited from a cohort with ACL injury 16 years earlier. Twelve uninjured males matched for age, sex, BMI and activity level served as a reference group. Self-reported patient-relevant data were obtained by the KOOS questionnaire.

**Results:**

There were no significant changes in knee stiffness during gait and step activity after training. For the cross-over hop, increased peak knee flexion during landing (from 44 to 48 degrees, p = 0.031) and increased internal knee extensor moment (1.28 to 1.55 Nm/kg, p = 0.017) were seen after training, indicating reduced knee stiffness. The KOOS sport and recreation score improved from 70 to 77 (p = 0.005) and was significantly correlated with the changes in knee flexion during landing for the cross-over hop (r = 0.6, p = 0.039).

**Conclusion:**

Knee-specific training improved lower extremity kinetics and kinematics, indicating reduced knee stiffness during demanding hop activity. Self-reported sport and recreational function correlated positively with the biomechanical changes supporting a clinical importance of the findings. Further studies are needed to confirm these results in women and in other ACL injured populations.

## Background

The anterior cruciate ligament (ACL) plays a major role in maintaining normal knee function. Injuries to the ACL are treated with training without surgery [1-3] or, more commonly, in combination with surgery [4-7]. The training usually emphasises normalisation of bilateral symmetries in joint mobility, neuromuscular control, muscle strength, and functional activity [8-12]. Neuromuscular training aims to enhance unconscious motor responses by stimulating both afferent signals and the central mechanisms responsible for dynamic joint control [13]. Neuromuscular training has become increasingly important and now plays a major role in the rehabilitation of ACL injuries. Most neuromuscular training programmes include balance exercises, dynamic joint stability exercises, plyometric exercises, and sport-specific exercises including balance and jump movements. Each component of the neuromuscular rehabilitation programme should be closely monitored by the physiotherapist, with corrections and feedback to improve dynamic control of the knee. Rehabilitation programmes including neuromuscular training are more effective in achieving good knee function and knee stability than rehabilitation programmes without neuromuscular training [3,12,14].

To monitor joint biomechanics during performances such as gait, step activity or hopping, the use of kinetics and kinematics has increased. The most severely affected parameters, when analysing lower extremity kinetics and kinematics in ACL-injured subjects, are knee flexion angle and internal knee extensor moment [15-19]. Decreased knee flexion angle and decreased internal knee extensor moment contribute to increased knee stiffness during loading. Increased knee stiffness shifts the load from the knee to the hip, foot and ankle [19-23]. The knee stiffening strategy seen in ACL deficient subjects may reflect the early stages of motor skill acquisition since as the skills level improves, knee stiffening decreases [24]. Increased knee stiffness, a load strategy frequently seen in ACL-injured subjects, is associated with excessive joint contact force [19]. Excessive joint contact force, which is manifested by an increased vertical ground reaction force (VGRF), may, in turn, lead to the development of knee OA [25]. This ought particularly to apply if the increased knee stiffness remains long after the injury and is present not only in the early stages of motor skill acquisition.

Dynamic stability of the knee joint depends on the ability to react quickly to sudden situation's changes. Functional joint instability may develop due to the lesion of mechanoreceptors in the joint capsule and ligaments in and surrounding the joint [26]. Maintaining dynamic stability during different skills off movements is dependent on cortically programmed muscle activations and reflex-supplied muscle contractions [27]. ACL-injured subjects have significantly slower reaction times and processing speeds than non-injured subjects [28] and thus neuromuscular training have the potential to improve muscular reaction time and joint position sense leading to improved knee kinetics and kinematics.

There is a lack of studies demonstrating the possible impact on lower extremity kinetics and kinematics due to training in subjects with an ACL injury.

We hypothesised that knee-specific training focusing on neuromuscular control would decrease the knee stiffness loading strategy commonly seen in these subjects. The aim of this study was therefore to determine if knee stiffness during loading, defined as changes in knee kinetics and kinematics of gait, step activity and cross-over hop, could be improved by a knee-specific 12-week supervised training programme.

## Methods

### Subjects

The subjects in this study were identified in 1989 [29]. All of them were males and suffered an ACL injury in 1986 while playing soccer and have been followed up three times since identification [29,30]. Of the 122 subjects who consented to have radiographs taken in the follow-up in 2000, those 25 living within a 2-hour drive of Lund University Hospital in the southern part of Sweden were invited to enter the present study. Of the eligible subjects, 12 accepted to participate. Nine subjects did not respond to the invitation and four declined to participate. The mean age of the participating subjects was 40 years, Table 1. The non-participating subjects were significantly younger (38 vs. 40 years, p = 0.05). No other significant differences were seen between the participants and non-participants. The current activity level ranged from Tegner level 2 to 9. Tegner activity level 2 represents recreational golf, cycling or swimming, working with small children or working as a waiter, while Tegner activity level 9 represents competitive soccer. Five of the twelve participating subjects had undergone ACL reconstruction at 0 to 4 years after the initial injury. In 2/5 cases a patella bone-tendon-bone autograft was used and in 3/5 cases an iliotibial band (ITB) autograft was used. When using the ITB autograft, a mean circular graft diameter of 8 mm was obtained using a 4- to 6-cm-wide and 18-cm-long strip of the anteroseptal part of the ITB (including part of the vastus lateralis fascia) [31]. The reasons behind the choice of graft were not recorded. One subject had undergone a suture of the ACL. Three of the participating subjects had radiographic OA in their injured knee. Radiographic OA was defined as grade 1 joint space narrowing combined with osteophytes or grade 2 joint space narrowing or more [32], corresponding to Kellgren and Lawrence knee OA grade 2 [33].

### Matched reference group

A previously described uninjured reference group [34], matched for age, BMI and activity level to the current study group, was used to determine whether any changes in kinetics and kinematics due to the intervention in the study group were in the direction of the reference group values or not. The mean age in the reference group was 39 years, Table 1. All participating subjects signed an informed consent form and the Ethics committee at the Medical Faculty, Lund University, approved the study.

### Design and intervention

All ACL-injured subjects were evaluated before and after 12 weeks of knee-specific training by motion analysis, using the VICON system, during gait, step activity and cross-over hop. The subjects in the reference group were evaluated at baseline only. To monitor the possible clinical changes from the training program the Knee injury and Osteoarthritis Outcome Score (KOOS) questionnaire was used. Isokinetic strength was evaluated to help interpret if the possible biomechanical changes were due to improvement in strength from the neuromuscular exercise programme.

**Figure 1:**
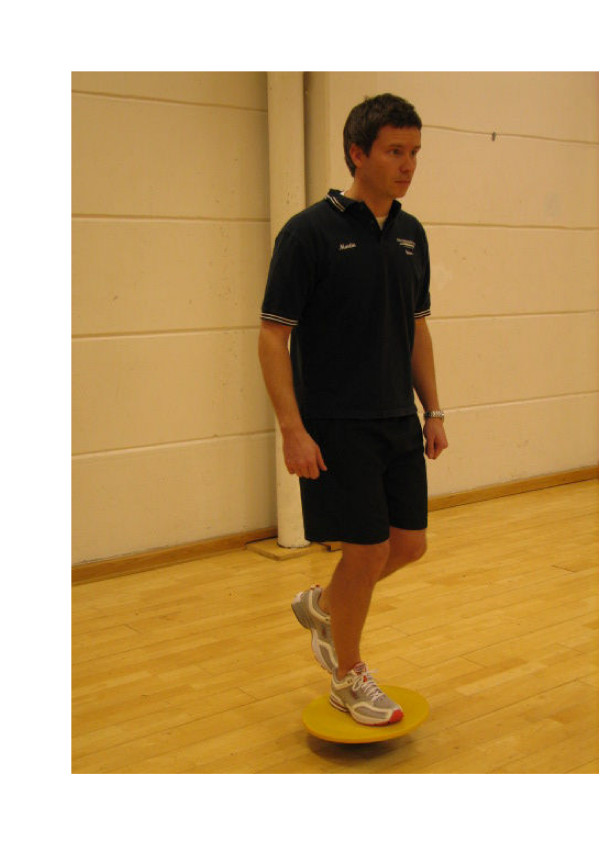
Balancing on one leg on a balance board.

**Figure 2:**
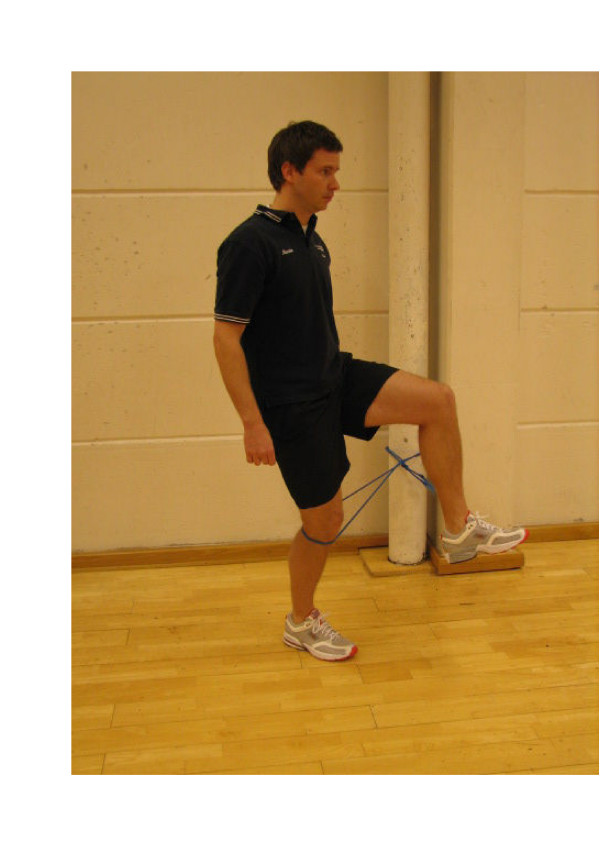
Knee control of the weight bearing leg with rubber band resistance.

**Figure 3:**
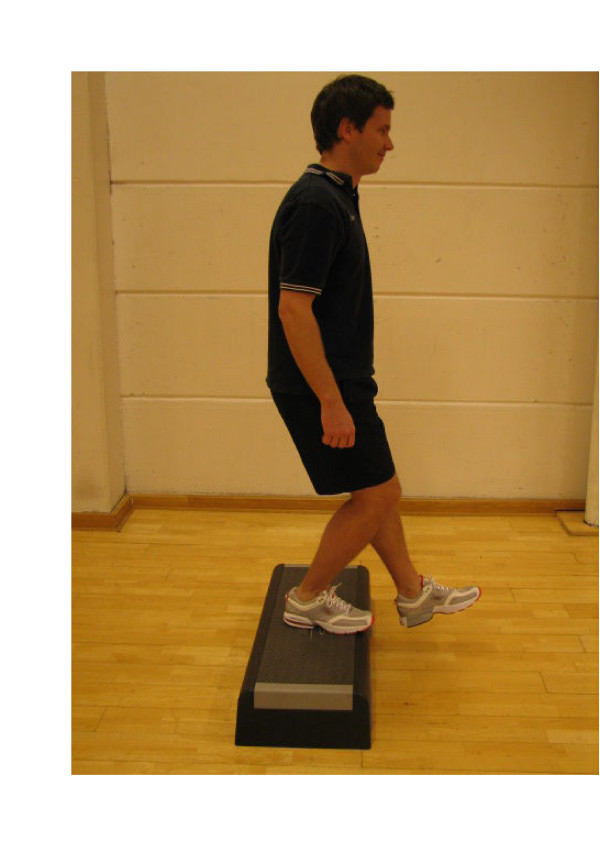
Stepping forward down from a step board with knee control on both the "supporting knee" and the "step down knee".

**Figure 4:**
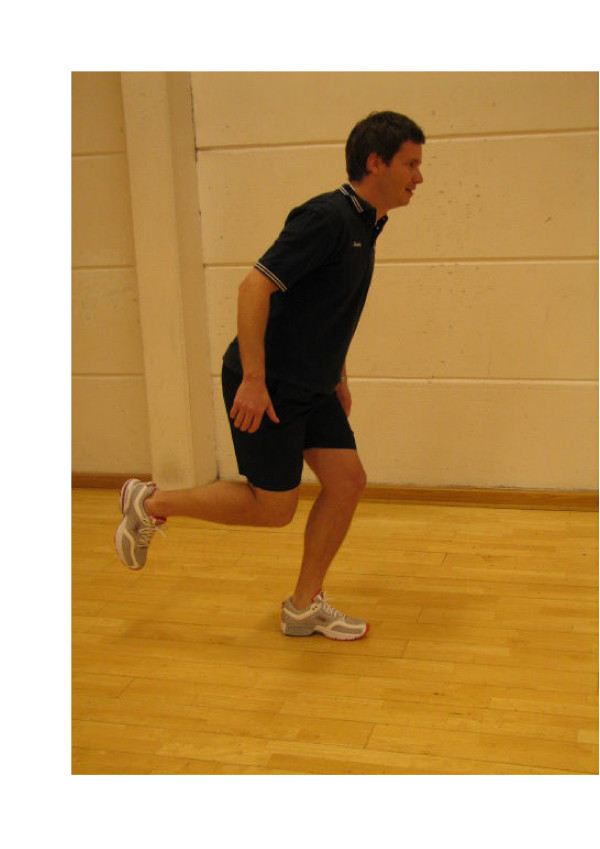
Jumping on one leg with knee control and knee elasticity during landing.

**Figure 5:**
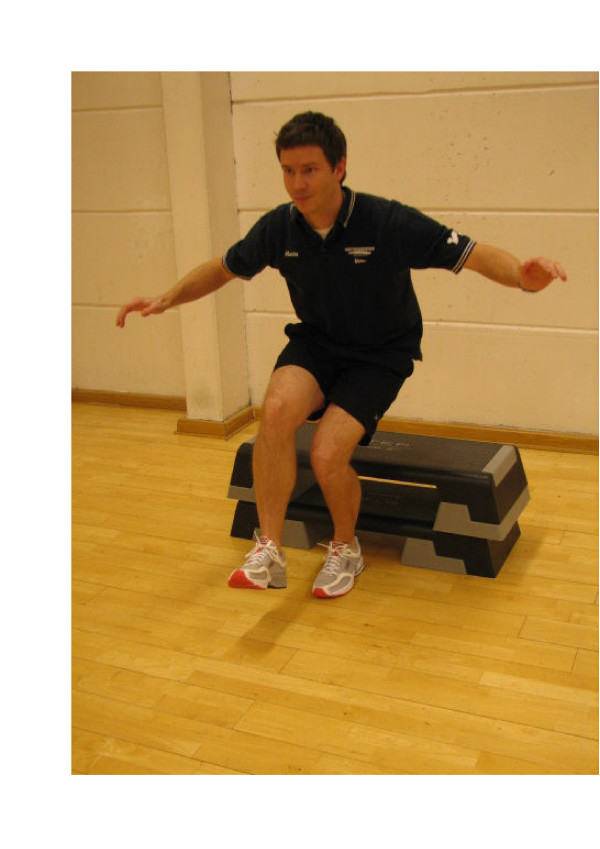
Rising up and sitting down on one leg.

A physiotherapist (AvP) supervised one-hour knee-specific sessions once a week for twelve weeks. In addition to the group training, each individual received instructions on home exercises. The training programme was based on clinical experience and a literature search and consisted of exercises focusing on neuromuscular control including: hop, knee control, balance, and core stability exercises [35-39]. Examples of exercises are given in Figure 1, 2, 3, 4, 5, 6. Each exercise was adapted to the subject's functional capacity. The difficulty was gradually increased from double to single leg exercises and from stable to unstable surfaces when tolerated. During the supervised sessions patients were repeatedly encouraged to maintain symmetry of the double-legged exercises throughout the performance and to focus on foot and knee placement during balance and jump exercises. Furthermore, subjects were told to use more knee and hip flexion during landing and take-off when practising jumping. The knee control exercises were performed in front of a mirror to make the subjects aware of the knee position during the performance. Finally, the core stability exercises were aimed at improving postural control.

The home exercises were almost identical to the supervised programme. Instead of using a step board for knee bending or jumping exercises, the subjects were instructed to use a staircase, and instead of using a pulley machine during knee control exercises they used a rubber band. All subjects were instructed to perform the home exercises once or twice a week. After the 12-week training period, all the subjects self-reported compliance with the home exercises by a questionnaire.

### Motion analysis

Lightweight surface markers were attached directly to the skin of the subjects and placed on standardised anatomical landmarks according to the biomechanical model of Kadaba et al. [40] and Davis et al. [41]. Marker position data were captured by a VICON 612 (OMG, Oxford, UK) system. This is a 3-dimensional passive marker motion capture system consisting of six cameras with a sampling frequency of 50 Hz, one data station and one PC on which the information is gathered and processed. Ground reaction force data were collected from one AMTI force plate (*OR6-7, Advanced Mechanical Technologies*). The size of the force plate was 505 × 465 mm and the sampling frequency 200 Hz. The marker positions were used, along with estimates of the joint centre locations and data from the force plate, to calculate the subject's 3-dimensional joint kinetics and kinematics. Calculation methods and model assumptions were as described [40-42]. The reliability of the kinetics and kinematics during gait and running is good [43,44]. The reliability of kinetics and kinematics during step and hop activity has not been assessed.

Three trials of each of the following activities for the right and left sides, respectively, were performed with a three-minute break between activities; 1) free-speed walking, 2) ascending and descending a 25-cm high step (step activity), and 3) cross-over hop test on one leg. In all tests the right leg was tested first and the order of the tests was the same for all subjects [34].

#### Gait

The subjects walked at a self-selected, comfortable speed on a 10 m walkway. A force plate embedded in the floor was located 3.5 metres after the start. Data from at least six dynamic trials, with three clean force plate strikes from each side, were captured.

#### Step activity

The front edge of the step was positioned approximately 60 cm from the force plate. The subject stood facing the step at a self-selected distance and was told to step up with one leg (referred to as the "supporting limb") and cross over the step with the opposite limb (referred to as the "step-over limb") [19]. It was the "step-over limb" that landed on the force plate.

#### Cross-over hop

The cross-over hop test was performed on a 6 m long course where the subject hopped from side to side across a 15 cm marking strip on the floor. The subject hopped three times in succession on the same foot, crossing the centre strip on each hop [10]. The first landing was a force plate strike.

### Isokinetic strength testing

The strength of each leg was evaluated with an isokinetic dynamometer (Cybex II Dynamometer 325, Lumex Inc., Ronkonkoma, NY). The subject was secured to the apparatus with straps across the chest, pelvis, thigh and ankle, according to the Cybex manual [45]. The subject was sitting with the thigh supported, with 90° hip flexion and the arms folded. The centre of motion of the lever arm was aligned as accurately as possible to the slightly changing flexion-extension axis of the knee joint, and the resistance pad was placed approximately 3 cm above the lateral malleolus on the tibia. The range of motion of the knee joint was set at 0–100°. The test-retest reliability of isokinetic muscle strength testing is good [46,47]. In order to familiarise the subjects with the operation of the dynamometer before formal testing began, they were allowed several sub-maximum practise efforts, after which three consecutive maximum efforts for knee extension and flexion at angle velocities of 60°/sec were recorded. A 20 sec rest was allowed between the sets. The peak torque (Nm) of the knee extension and flexion muscle strength was recorded.

### Self-reported outcomes

The Knee injury and Osteoarthritis Outcome Score (KOOS) was used to evaluate self-reported pain, function and quality of life [48,49]. The KOOS consists of five subscales; Pain, Symptoms, ADL, Sport and Recreational function, and knee-related QoL, which are scored separately on a 0–100, worst-to-best, scale. The Tegner activity level scale, scored from 1–10, where 10 indicates professional soccer, was used to evaluate the current activity level [50]. Both instruments have good validity and reliability for subjects with ACL injuries [51,52].

### Data management

Knee kinetics and kinematics data were obtained from graphs. To avoid bias, the data were read in a blinded fashion. All legs were given a number from a random number list. This list was handled by the last author and was not available to the graph reader. Kinetic and kinematic data for gait were calculated across the stance phase and swing phase and normalised to 100% of the gait cycle. A gait cycle starts at heel-strike and stops at the next heel-strike by the same foot [53]. Kinetic and kinematic data for step and hop activity were calculated across the stance phase and normalised to 100% of stance. Calculated kinetic data were normalised for body mass in kilograms. The step activity data was normalised for leg length, as measured from the spina iliaca anterior superior (SIAS) to the medial malleolus. All modelling assumptions are based on the work by Ramakrishnan et al. [42]. At the time of processing, all three trials were calculated and a mean of the three trials was used for analysis.

#### Walking

The kinematic variable studied was peak knee flexion during loading response, and the kinetic variables were internal knee extensor moment at peak knee flexion and the peak vertical ground reaction force (VGRF) during the first 25% of the gait cycle.

#### Step activity

The kinematic variable studied was knee flexion of the supporting limb, obtained when the step-over limb landed on the force plate. The kinetic variables were internal knee extensor moment at peak knee flexion and the peak vertical ground reaction force (VGRF) of the step-over limb when landing on the force plate.

#### Cross-over hop

The kinematic variable studied was knee flexion during landing. The kinetic variables studied were internal knee extensor moment at peak knee flexion, and the peak vertical ground reaction force (VGRF) of the first jump when landing on the force plate.

### Statistics

Results are given as mean ± SD unless otherwise stated. The data were analysed using the Statistical Package for Social Sciences for Windows (SPSS Version 12.0.1). The results had a normal frequency distribution, allowing the use of parametric statistics. We found no differences in interpretation when employing parametric or non-parametric statistics. The paired samples t-test was used to determine levels of significance when comparing the groups before and after training. Pearson's correlation coefficient was used to determine the correlation between knee extensor strength and knee extensor moment, and to determine the correlation between the changes in knee kinetics/kinematics and the change in self-reported Sport and Recreational function according to the KOOS questionnaire. The level of significance for statistical measures was set a priori to *p *≤ 0.05 and all tests were one-tailed.

## Results

### Compliance with intervention and self-reported outcomes

Ten ACL-injured subjects took part in all twelve supervised sessions while two subjects took part in only four sessions for work-related reasons. The home exercises were performed once or twice weekly by 11 of the subjects. One subject only participated in the supervised sessions. In total, the number of completed training sessions, both supervised and non-supervised home training, averaged twice a week, and ranged from 1–3 times/week.

**Figure 7:**
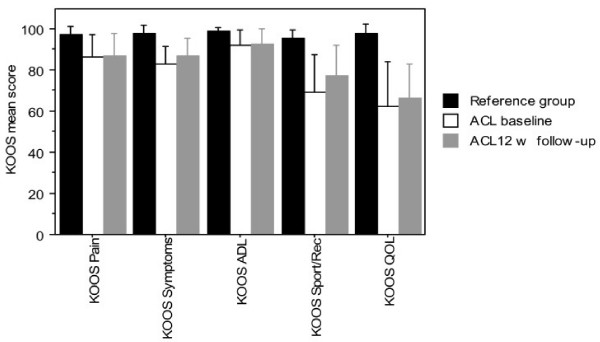
Mean (95% CI) KOOS scores for the reference group and the ACL group before and after the 12 week training programme.

To assess the clinical effect of the training self-reported outcomes were assessed. While no change was seen in pain or symptoms, a significant improvement was seen in the KOOS subscale Sport and Recreational function (70 to 77, p = 0.05), Figure 7.

To the question: "Would you resume the training programme if your knee got worse?", 11 subjects replied "yes" while one replied "I don't know".

### ACL-injured group before and after training

The training program introduced changes in kinetics and kinematics making the ACL injured subjects more similar to the reference group (Table 2). The most indicative test situation was the cross-over hop, in which peak knee flexion during landing and internal knee extensor moment changed significantly after training, p < 0.015. Peak knee flexion during landing increased from 44 degrees before training to 48 degrees after training and approached the reference group with a mean peak knee flexion during landing of 49 degrees. The internal knee extensor moment during cross-over hop also increased after training from 1.28 to 1.55 Nm/kg, p = 0.017. The VGRF during cross-over hop did not change significantly after training. The changes in biomechanics during the cross-over hop were positively correlated (knee flexion during landing r = 0.6, p = 0.019, and knee extensor moment r = 0.3, p = 0.151) to the improvement in self-reported Sport and Recreational Function.

**Figure 8:**
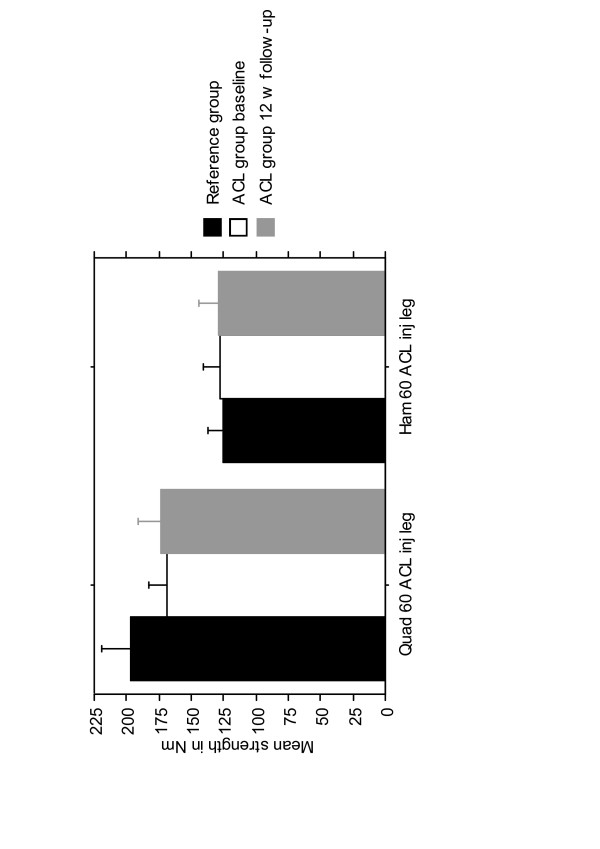
Mean (95% CI) isokinetic thigh strength at 60 degrees/second for the reference group and the ACL group before and after the 12 week training programme.

The aim of the training program was to improve neuromuscular control and no specific strength exercises were performed. To assure that the possible effect seen from the intervention was due to neuromuscular improvement and not strength, isokinetic strength was evaluated. There were no significant differences in any of the isokinetic strength measures when comparing controls, ACL subjects at baseline and after 12 weeks of training, Figure 8. Isokinetic quadriceps strength at 60 degrees per second showed poor correlation with the improvement in internal knee extensor moment during gait, step activity and cross-over hop; r = 0.03, 0.19 and -0.06, respectively, p > 0.3.

## Discussion

This study indicates that neuromuscular training may introduce changes in the loading pattern of the affected leg in ACL injured subjects that are measurable by 3-dimensional motion analysis. Our study group was very homogenous with regard to some aspects; they were all mid life males who sustained an ACL injury during soccer play 16 years ago, but heterogeneous with regard to current joint status. The study group consisted of only 12 subjects and although a similar direction of change was seen for all three evaluated functions, the changes were only statistically significant during the most demanding function, the cross-over hop, indicating that the t-test comparisons may be limited by the small number of subjects. Another limitation of the study may be that there were no significant differences between the ACL-injured subjects and the controls at baseline [34]. Thus our data should be viewed as preliminary and interpreted with caution. 3-dimensional motion analysis is a feasible method for evaluation of changes due to neuromuscular training and should be employed in future larger studies that aim to generalize these findings also to women and other ACL injured populations.

The few available studies using biomechanical outcomes to evaluate the impact of neuromuscular training have used interventions similar to those used in the present study, and similarly show improvement in joint flexion angles during gait and hop activities. Myer and co-workers studied "Drop Vertical Jump" and "Medial Drop Landing" tests to analyse the abduction angles and moment in the hip, knee and ankle. Both aspects were improved by neuromuscular training [36]. Chmielewski et al. showed that perturbation training of ACL-deficient potential copers improved knee stiffness during gait, defined as knee flexion and co-contraction index. This change was in the direction of uninjured control subjects [23].

Most studies using neuromuscular training have evaluated the effect on injury prevention [35,54-60]. Studies of neuromuscular training, in comparison to traditional strength training, in ACL injured subjects have shown better result in sensory outcomes such as proprioception, motor outcomes such as strength and functional tests, and self-reported outcomes [12,14]. A prospective, double-blind, randomised, clinical trial in subjects with an on average one year old ACL injury compared a programme for muscle strength with a neuromuscular programme. The investigators found a significantly greater improvement in both proprioception and functional score in the neuromuscular group after 12 weeks' training [14]. Significantly better results, evaluated by one leg hop test, strength test and self-reported questionnaires, were found after supervised neuromuscular training compared with self-monitored training in non-reconstructed ACL subjects with acute injuries [12]. In the present study, the movement pattern was still amenable to knee-specific training, indicating that knee-specific training is feasible also for individuals 16 years past the injury. The poor correlation between quadriceps strength and knee extensor moment seen in the present study supports the theory that lower extremity kinetics and kinematics could be influenced by training focusing on neuromuscular control only without special emphasis on improving lower extremity muscle strength [12,14].

The optimum training period or number of training sessions to improve the lower extremity loading pattern is difficult to define, largely due to differences in the subjects' body awareness and ability to respond to feedback given to improve neuromuscular control. This study showed that duration of 12 weeks and a frequency of twice a week were sufficient to decrease knee stiffness.

## Conclusion

Despite subjects being 16 years post-injury, knee-specific training improved lower extremity kinetics and kinematics, indicating reduced knee stiffness and significantly so during the more demanding hop activity. Moreover, self-reported sport and recreational function correlated positively with the biomechanical changes indicating a clinical importance of the findings. Further studies are needed to confirm these results in women and in other ACL injured populations.

## Competing interests

The author(s) declare that they have no competing interests.

## Authors' contributions

AvP conceived and designed the study, acquired the data, conducted data analysis and drafted the manuscript. EMR contributed to the conception and the design of the study and reviewed the manuscript critically. MH contributed to the design of the study and reviewed the manuscript critically. EH reviewed the manuscript critically. All authers read and approved the final manuscript.

## Pre-publication history

The pre-publication history for this paper can be accessed here:



## Figures and Tables

**Figure 6:**
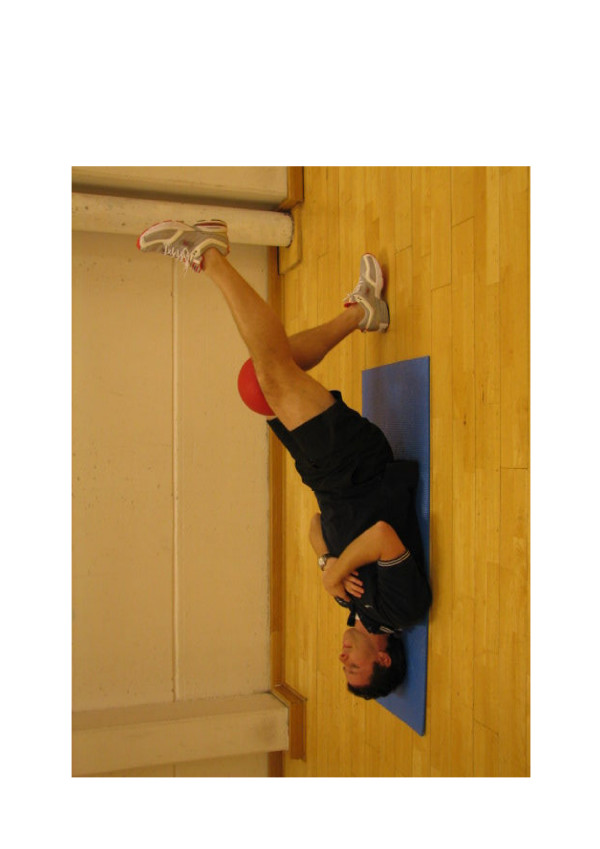
Core control while lifting pelvis and lower back on single leg with simultaneous contraction of hip adductors and buttocks.

**Table 1:** Characteristics of the participants.

	Reference group	ACL group
Age (year)	39 ± 6 (32–53)	40 ± 5 (32–53)
Weight (kg)	81 ± 5 (72–92)	80 ± 8 (70–100)
Height (cm)	181 ± 4 (175–187)	182 ± 4 (173–187)
Leg-length injured leg (cm)	96 ± 3 (92–100)	95 ± 3 (87–101)
Leg-length non-injured leg (cm)	96 ± 3 (92–100)	95 ± 4 (88–103)
BMI	24.5 ± 2 (22.0–26.5)	24.5 ± 2 (22.1–30.3)
Tegner Activity level	5.5 ± 4 (3–8)	5.5 ± 3 (2–9)

Characteristics of the ACL injured group and the reference group (mean ± SD (range)). For the Tegner Activity level median ± IQR is given.

**Table 2:** Kinetics and kinematics during gait, step activity and cross-over hop.

	Reference group Corresponding side	ACL group injured side at baseline	ACL group injured side at 12 w follow-up	P-value
**GAIT**				
VGRF (BW) ^a^	1.09 ± 0.1	1.12 ± 0.1	1.12 ± 0.1	0.448
Peak knee flexion at loading response (degrees)	17 ± 3	18 ± 4	18 ± 3	0.097
Knee extensor moment (Nm/kg), (internal moment)	0.42 ± 0.1	0.44 ± 0.2	0.49 ± 0.2	0.197
**STEP ACTIVITY**				
VGRF (BW) ^b^	1.59 ± 0.2	1.66 ± 0.1	1.58 ± 0.2	0.119
Peak knee flexion of supporting limb (degrees)	52 ± 6	48 ± 9	50 ± 9	0.130
Knee extensor moment (Nm/kg), (internal moment)	0.56 ± 0.2	0.44 ± 0.3	0.54 ± 0.2	0.084
**CROSS-OVER HOP**				
VGRF (BW) ^b^	1.88 ± 0.1	1.86 ± 0.2	1.85 ± 0.2	0.337
Peak knee flexion during landing (degrees)	49 ± 7	44 ± 6	48 ± 5	0.015
Knee extensor moment (Nm/kg), (internal moment)	1.49 ± 0.4	1.28 ± 0.5	1.55 ± 0.6	0.008

a) VGRF in gait was measured during the first 25% of the gait cycle. b) VGRF in step and cross over hop were measured during the initial contact with the force plate of the step over limb.

Mean ± SD for kinetics and kinematics during gait, step activity, cross-over hop for the reference group and the ACL group before and after the 12 week training programme. The paired samples t-test was used to compare the ACL group at baseline and at the 12 week follow-up.
